# Dissociative Ionization and Coulomb Explosion of Molecular Bromocyclopropane in an Intense Femtosecond Laser Field

**DOI:** 10.3390/molecules23123096

**Published:** 2018-11-27

**Authors:** Botong Liu, Yan Yang, Haitao Sun, Zhenrong Sun

**Affiliations:** 1 State Key Laboratory of Precision Spectroscopy, School of Physics and Materials Science, East China Normal University, Shanghai 200062, China; liubotong1993@foxmail.com (B.L.); htsun@phy.ecnu.edu.cn (H.S.); zrsun@phy.ecnu.edu.cn (Z.S.); 2 Collaborative Innovation Center of Extreme Optics, Shanxi University, Taiyuan 030006, China

**Keywords:** bromocyclopropane (BCP), femtosecond laser field, dissociative ionization (DI), Coulomb explosion (CE), dehydrogenation, dissociation time

## Abstract

The dissociative ionization and Coulomb explosion of molecular bromocyclopropane (BCP) has been experimentally investigated by time-of-flight mass spectrum and dc-slice imaging technology. The sliced 2D images, kinetic energy releases and angular distributions of the fragment ions are obtained under the intense femtosecond laser fields (8.0 × 10^13^–2.0 × 10^14^ W/cm^2^). The results indicated that the low kinetic energy release (KER) components come from dissociative ionization of BCP^+^, while the high KER components come from Coulomb explosion of BCP^2+^. The chemical reaction path of BCP^+^ has been calculated by ab initio calculation, furthermore, the C-Br bond cleavage involved Coulomb explosion channels have been revealed, and the corresponding dehydrogenation mechanism has been confirmed.

## 1. Introduction

With the development of the ultrafast laser technique, the molecular dynamic process in the intense femtosecond laser field has attracted extensive attention [1,2,3]. As well known, the molecule exposed in an intense femtosecond laser field will be ionized firstly and then dissociate into several parts along different chemical bond(s). According to the Keldysh theory [4], in the case of low laser intensity, it’s believed that multiphoton absorption dominates the ionization process, and the parent cation usually dissociates into fragment ions and neutral parts with relative low kinetic energies, which is called dissociative ionization (DI). In the case of high laser intensity, however, the electrons can be directly stripped away by the intense laser field and the multi-charged parent molecular ions would dissociate into several parts by coulomb repulsive force, which is called Coulomb explosion (CE). Generally, the exploded fragments have much higher kinetic energies than those from DI process, in addition, the ejection direction of the ions originating from CE channel has a strong relationship with the direction of laser polarization. By analyzing the momentum distribution, energy information and angular distribution of the fragment ions, the information about excited states of the highly charged molecular ions, dynamic evolution of the electronic wave packet on different potential energy surfaces, and the reconstruction of the complicated molecule system can be obtained and achieved [5,6,7].

Due to the importance in the field of atmospheric physics and environment chemistry, plenty of studies have been focused on the carbon halogen dissociation by single-photon excitation using ultraviolet laser field [8,9,10,11]. H.K. Kim et al. have investigated the photodissociation of bromocyclopropane at 234 nm laser field using ion-imaging technique with the REMPI (Resonance-Enhanced Multiphoton Ionization) scheme. They infer that the non-adiabatic transition probability from the 3A′ potential energy surfaces was estimated to be 0.90 [12]. S. Pandit et.al. studied the photodissociation of bromocyclopropane in its A-band by the violate excitation (230–267 nm) and proposed three dissociative pathways, in which the C–Br bond fission and the cyclopropyl ring opening are involved [13]. However, the ionization and dissociation of molecular BCP is rarely investigated under the intense femtosecond laser field. In this paper, we experimentally demonstrate the photodissociation process of bromocyclopropane molecules in the near-infrared (800 nm) intense femtosecond laser field by a dc-sliced ion velocity map imaging technique. By analyzing the kinetic energy releaser and angular distributions of the fragment ions, we infer that the low KER components come from dissociative ionization of BCP^+^, while the high KER components come from Coulomb explosion of BCP^2+^. Besides, the corresponding dissociative reaction path is calculated by GAUSSIAN 16 software packages. Furthermore, the C-Br bond cleavage involved Coulomb explosion channels have been revealed, and the broadening of the angular distribution for C_3_H_3_^+^ ions is quantitatively analyzed.

## 2. Experiment and Computational Methods

The experiment is performed by our home-made dc-slicing ion imaging system, which has been described elsewhere in detail [14]. Briefly, the BCP sample bubbled by helium is ejected into the reaction chamber by a pulsed valve (general valve, Parker) with a repetition rate of 100 Hz and the duration time of 170 us. Thus, the background pressure of the reaction chamber is varied from 4.0 × 10^−9^ mbar to 7.4 × 10^−8^ mbar. A linear polarized femtosecond laser pulse (*50 fs full width at half maximum (FWHM), center-wavelength 800 nm, repetition rate of 1 KHz*) interacts with the supersonic BCP molecular beam at a right angle by a biconvex lens with a 40 cm focal length. The produced fragment ions guided by electric field pass through the dc-slicing apparatus with multi-lens, where the momentum focusing is maintained and every individual ion cloud is stretched to meet the slice condition. Afterwards, these ions are detected by a two-stage microchannel plates (MCPs) coupled with a P47 phosphor screen. The 2D momentum images of each fragments are the central slice (around *p_z_*= 0) of the corresponding 3D momentum distribution and are obtained by a charge coupled device camera (PI-MAXII, Princeton Instrument) with 5 ns time resolution. the system timing sequence control is performed by a Stanford Instrument Digital Delay Pulse Generator (DG645). The laser pulse polarization vector is perpendicular to the time-of-flight axis and the laser intensity in the focal volume is estimated from 8.0 × 10^13^ to 2.0 × 10^14^ W/cm^2^, which is calibrated by the Ar^2+^/Ar^+^ yield ratio proposed by Guo et al. [15].

To better illustrate the dissociation process of the BCP^+^ ions in the intense femtosecond laser fields, the related dissociative pathways are calculated by the Gaussian *16* software package [16], and the relate calculations are carried out at the G4 level [17]. In our calculation, the ionization energy of the BCP molecule is 9.52 eV, which is nearly same as the value (9.53 eV) in Ref [18]. The great agreement between our calculations and previous experiment results can demonstrate the feasibility of our theoretical methodology used in this paper.

## 3. Results and Discussion

### 3.1. Time-of-Flight Mass Spectrum and Dc-Sliced Images

**Figure 1:**
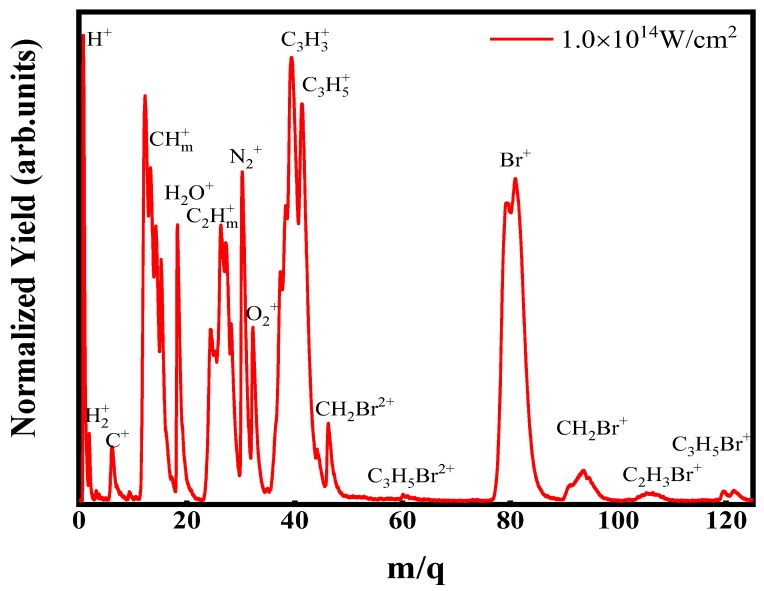
The mass spectrum of BCP molecule irradiated by the femtosecond laser field with the intensity of 1.0 × 10^14^ W/cm^2^ at 800 nm.

Figure 1 shows the TOF mass spectrum of molecular BCP irradiated by the femtosecond laser field with the intensity of 1.0 × 10^14^ W/cm^2^. Clearly, except the parent ion C_3_H_5_Br^+^, the main fragment ions H^+^, H_2_^+^, C^2+^, CH_m_^+^, C_2_H_m_^+^, C_3_H_3_^+^, C_3_H_5_^+^, Br^+^, CH_2_Br^+^ and C_2_H_3_Br^+^ (*m* = 0–3) can also be observed. Due to the isotopes of ^79^Br and ^81^Br, all the fragment ions containing bromide atom exhibit multi-peak structures. It is noted that the cleavage of C-Br and C-C bond predominates the dissociation process under our experiment condition, therefore, the fragmentations are mainly attributed to the dissociation of singly charged parent ions BCP^+^ and Coulomb explosion of doubly charged parent ions BCP^2+^ along these two bonds. To further investigate the related dissociation processes, the dc-slice imaging technology is utilized to measure the kinetic energy releases (KER) and angular distributions (AD) of these fragment ions.

**Figure 2:**
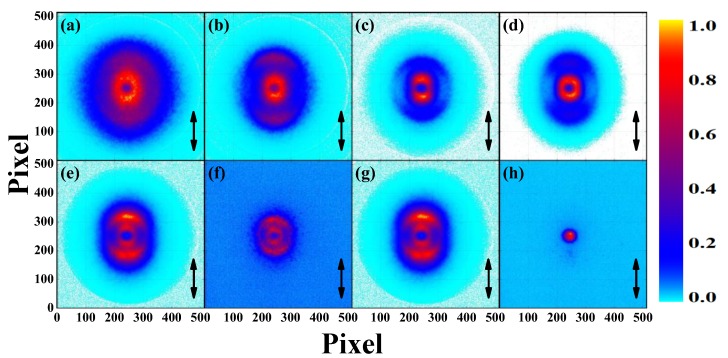
The sliced images of the ions (**a**) CH_2_^+^, (**b**) C_2_H_3_^+^, (**c**) C_3_H_3_^+^, (**d**) C_3_H_5_^+^, (**e**) Br^+^, (**f**) CH_2_Br^+^, (**g**) C_2_H_3_Br^+^ and (**h**) C_3_H_5_Br^+^ with the laser intensity of 1.0 × 10^14^ W/cm^2^ and the double black arrows represent the direction of laser polarization vector. The color map of each image is normalized to 1, individually.

**Figure 3:**
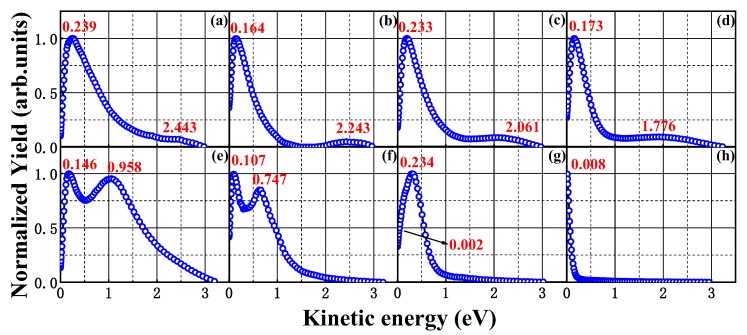
The corresponding kinetic energy release distributions of the fragment ions (**a**) CH_2_^+^, (**b**) C_2_H_3_^+^, (**c**) C_3_H_3_^+^, (**d**) C_3_H_5_^+^, (**e**) Br^+^, (**f**) CH_2_Br^+^, (**g**) C_2_H_3_Br^+^ and (**h**) C_3_H_5_Br^+^ with the same experiment condition as mentioned in Figure 2.

In Figure 2, the sliced images of the fragment ions (a) CH_2_^+^, (b) C_2_H_3_^+^, (c) C_3_H_3_^+^, (d) C_3_H_5_^+^, (e) Br^+^, (f) CH_2_Br^+^, (g) C_2_H_3_Br^+^ and (h) C_3_H_5_Br^+^ with laser intensity of 1.0 × 10^14^ W/cm^2^ are shown, and these ions are the main production for cleavage of C-Br and C-C bond. The black double arrow representing the direction of linear polarization vector of laser field is parallel to the detector plane. Figure 3 shows the corresponding KER distributions, where the KER peaks representing different dissociation channels are fitted by the Gaussian functions. According to our previous studies [6,19,20,21], the photo-dissociated ion fragments with low KER can be attributed to the dissociative ionization process, and those with high KER can be attributed to the Coulomb explosion process.

### 3.2. Dissociation of Singly Charged Molecular Ion BCP^+^ in 800 nm Femtosecond Laser Fields

Considering that the laser pulse duration is about 50 fs in our experiment and most single ionizations will take place in the first few cycles, it is believed that the dissociation process should be after the single ionization process. So the above-mentioned produce fragments with low KER should be attributed to the dissociative ionization of the parent molecule ions. The estimated reaction channels are described as follows

C_3_H_5_Br^+^→C_3_H_5_^+^+Br (1)

C_3_H_5_Br^+^→C_3_H_3_^+^+Br+H_2_ (2)

C_3_H_5_Br^+^→CH_2_Br^+^+ C_2_H_3_ (3)

C_3_H_5_Br^+^→C_2_H_3_^+^+ CH_2_Br (4)

C_3_H_5_Br^+^→CH_2_^+^+ C_2_H_2_Br (5)

C_3_H_5_Br^+^→C_2_H_3_Br ^+^+ CH_2_ (6)

C_3_H_5_Br^+^→Br ^+^+ C_3_H_5_ (7)

In order to further confirm above photodissociation channel assigning to the DI process, we theoretically calculate the appearance energy and available energy of these channels (1)–(7) by GAUSSIAN *16* software packages. The definition of the appearance energy and the available energy value of chemical reaction channel are expressed in Equation (1), where $\sum_{i}E_{i}$ and $\sum_{j}E_{j}$ represent the sum of the energy of the reaction products and reactants, respectively, and n represents the minimum number of photons in order to ensure the reaction take place. Generally, the larger the appearance energy is, the more difficultly chemical reaction is occurred. Notably, part of the available energy releases as kinetic energy of the fragment ions, thus, the total translational energy of each channel should be smaller than the related available energy.

(Equation (1)) $$E_{\mathrm{appearance}}=\underset{i}{\sum }E_{i}-\underset{j}{\sum }E_{j}\;E_{\mathrm{avail}}{\;=\;\mathrm{nh}\nu \;-\;E}_{\mathrm{appear}}$$

here, the electron recoil momentum is not considered in the calculation and only the ion-neutral partner is calculated. According to the Equation (2) and fragment KER (the values of the lower KER peaks in Figure 3), the transnational kinetic energy release (TER) of the corresponding photo-dissociation channel based on a center-of-mass coordinate can be obtained, where m_a_ and m_b_ represent the mass of reaction fragment a and b, respectively, and v_a_ is the velocity of reaction fragment a. The appearance energies, KER values of the fragment ions, tTER values and available energies of each channel (1)–(7) are listed in Table 1. Obviously, all the TER values of these channels are in the range of the calculated available energy, which further indicate that the low KER components of these fragment ions should result from the dissociation of BCP^+^.

(Equation (2)) $$\mathrm{TER}\;=\;\frac{1}{2}{(m}_{a}{\;+\;m}_{b})(\frac{m_{a}}{m_{b}}{)\nu }_{a}^{2}$$

**Figure 4:**
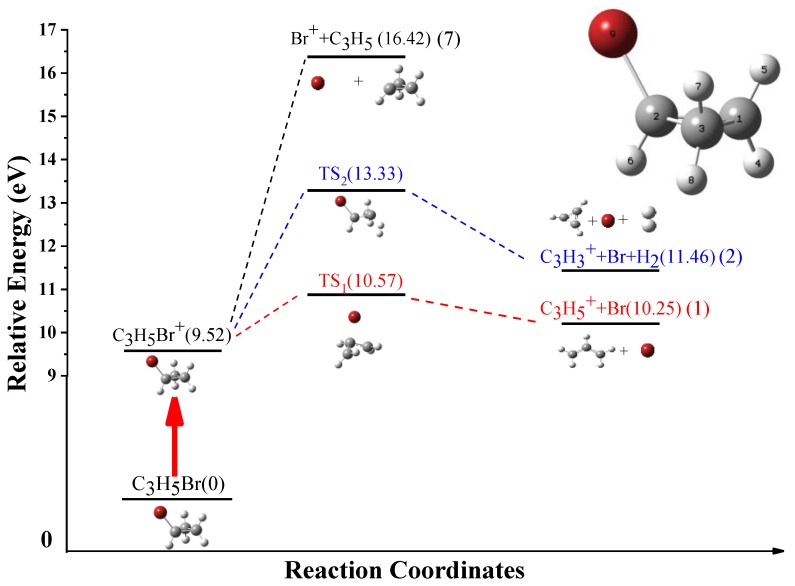
The dissociation pathways of the singly charged molecular ion BCP^+^. The calculation is performed at G4 level by Gaussian *16* program.

Figure 4 shows the calculated dissociation channel (1), (2) and (7) for the singly charged parent ions. For simplicity, the ground state energy of the molecular BCP is set as zero.

**Figure 5:**
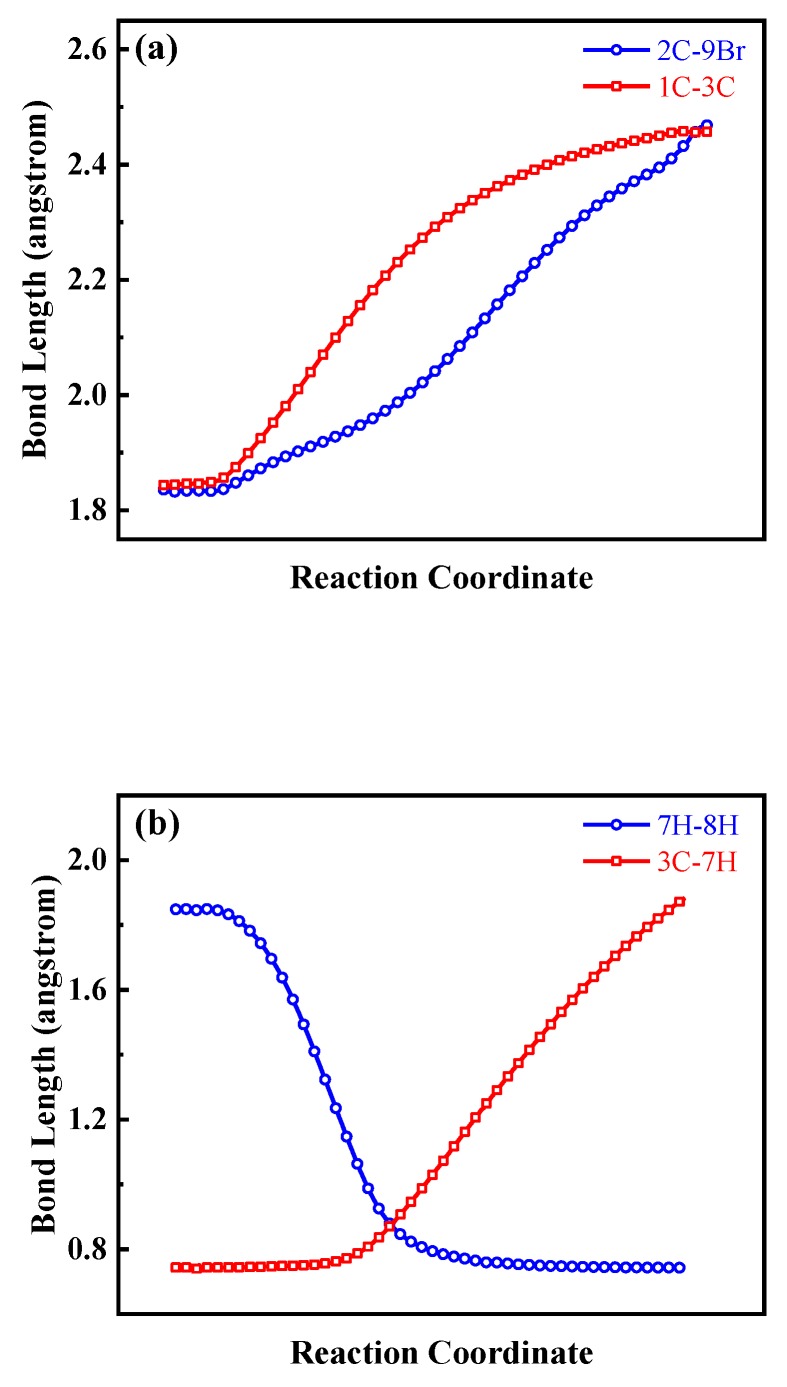
(**a**) The variations of 2C-9Br (blue circle) and 1C–3C (red square) bond length of single charged molecular ion alone the channel (1), (**b**) The variations of 7H–8H (blue circle) and 3C–7H (red square) bond length of single charged molecular ion alone the channel (2) in Figure 4, respectively.

For channel (1), the parent ions BCP^+^ dissociate along C-Br bond and pass through a transition state (TS_1_) with the energy barrier of 1.05 eV, and the alkyl group undergoes a ring opening process to form the C_3_H_5_^+^ and Br. As shown in Figure 5a, during the reaction, the bond length of 1C–3C bond increases from 1.85 to 2.45 Å, meanwhile, the other two C-C bonds length is nearly unchanged and the bond length of 2C-9Br bond keeps elongation till broken. This kind of structure deformation indicates that the ring opening dynamics is involved in our experiment unambiguously. However, in channel (7), where the same chemical bond is broken, the fragment C_3_H_5_ still maintains the ring structure until the 2C-Br bond is totally dissociated. Except the structure difference between fragment C_3_H_5_^+^ and C_3_H_5_, the appearance energy of channel (7) is much higher than that of channel (1). This can be explained by the difference of the ionization potential energy of the neutral fragment C_3_H_5_ and Br (6.92 and 13.09 eV, respectively), which is consist with our previous work [21].

For channel (2), the parent ions dissociate along the C-Br and C-H bond synchronously. The ions C_3_H_5_Br^+^ firstly pass through a transition state (TS_2_) with the energy barrier 3.81 eV then decompose into C_3_H_3_^+^, Br and H_2_. Obviously, as shown in Figure 5b, the two hydrogen atoms are from the same carbon atom 3C, and the distance between them decreases from 1.85 to 0.74 Å while the 3C-7H bond elongates from 0.75 to 1.88 Å during the reaction process. Here, it is based on the following consideration that the production of channel (2) is molecular H_2_ rather than two single H atoms. In one hand, the distance between these two hydrogen atoms have stabilized at 0.74 Å which is equal to the bond length of a neutral molecular H_2_ [22]. In the other hand, the bonding process of two hydrogen atoms is an exothermic process which means the production with molecular H_2_ has the lower energy and is more stable in chemical reaction.

### 3.3. Coulomb Explosion of Doubly Charged Molecular Ions BCP^2+^

For the high KER components in the dc-sliced images, it is believed they are mainly attributed from Coulomb explosion process. In the classic point charge model, the kinetic energy of a pair of fragment ions should meet the Equation (3)

(Equation (3)) $$\frac{E_{k}\left(A^{p+}\right)}{E_{k}\left(B^{q+}\right)}\;=\;\frac{m\left(B^{q+}\right)}{m\left(A^{p+}\right)}\;\;\;\Delta \;=\;\left|\frac{\mathrm{KER}\left(A^{p+}\right)/\mathrm{KER}\left(B^{q+}\right)-m\left(B^{q+}\right)/m\left(A^{p+}\right)}{m\left(B^{q+}\right)/m\left(A^{p+}\right)}\right|$$

where E_k_ is kinetic energy, m is the mass of the fragment ion, Δ represents the experimental error factor, and p, q denotes the charge state of the fragment ions, respectively. Table 2 shows the ratio $\mathrm{KER}\left(A^{p+}\right)/\mathrm{KER}\left(B^{q+}\right)$, $m\left(B^{q+}\right)/m\left(A^{p+}\right)$ and the experimental error factor Δ. Due to the limitation of the experimental condition and the artificial error introduced by data processing, the error factor Δ smaller than 5% indicates that the dissociative double ionization(DDI) process exists in our experiment. As a result, two main CE channels can be inferred as follows

C_3_H_5_Br^2+^→C_3_H_5_^+^+Br^+^ (8)

C_3_H_5_Br^2+^→C_3_H_3_^+^+Br^+^+H_2_ (9)

**Figure 6:**
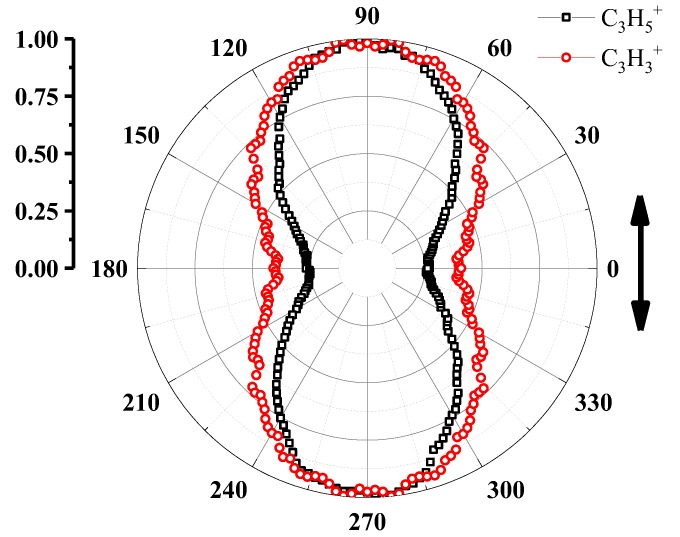
The angular distributions of C_3_H_5_^+^ and C_3_H_3_^+^ ions and the double arrow represents the direction of laser polarization.

For C_3_H_5_^+^ and Br^+^, it is clear that the C-Br bond is broken during the Coulomb explosion process, and the two exploded fragment ions meet Equation (3) quite well. However, for channel (9), the relate experimental error factor exceeds 5%, which should be given rise to the dehydrogenation process. Therefore, three possible mechanisms for the H atom(s) ejection are summarized as follow:

(1) pre-dehydrogenation: P→[P’]^2+^ + nH + 2e→A^+^ + B^+^ + nH + 2e^−^
(2) direct-dehydrogenation: P→P^2+^ + 2e→A^+^ + B^+^ + nH + 2e^−^
(3) post-dehydrogenation: P→P^2+^ + 2e→A^+^ + B^+^ + 2e^−^, B^+^ + mhν→C^+^ + nH

where P is the parent molecule, and A, B and C denote the DDI products. In pathway (1), the neutral hydrogen atom(s) has (have) been ejected before the typical two-body Coulomb explosion. If C_3_H_3_^+^ and Br^+^ are produced by the pre-dehydrogenation process, the KER relationship between C_3_H_3_^+^ and Br^+^ should satisfy Equation (3) as well as channel (8). In addition, if the pre-dehydrogenation governs the DDI process, the doubly charged molecular C_3_H_3_Br^2+^ should be observed in the mass spectrum and sliced images, however, the signal of C_3_H_3_Br^2+^ ions is not obtained at all during the measurement. In pathway (3), the photodissociation fragments produced by DDI will further dissociate into another ion and hydrogen atom(s). Considering the laser pulse duration (FWHM = 50 fs) in our experiment, it is nearly impossible for exploded products to further absorb photons then dissociate into several parts, therefore, the post-dehydrogenation process is eliminated. As a result, it is proposed that the direct-dehydrogenation process dominates our DDI process and the dehydrogenation indeed impacts the KERs and angular distributions of the fragments. For KERs, due to the ejection of hydrogen atoms, it is reasonable that the channel (9) cannot meet the Equation (3) very well. For angular distributions, as shown in Figure 6, the angular distribution of C_3_H_5_^+^ and C_3_H_3_^+^ ions share the similar anisotropic characteristic, which indicates C_3_H_5_^+^ and C_3_H_3_^+^ ions are mostly ejected along laser polarization direction, however, the dehydrogenation broadens the angular distribution of C_3_H_3_^+^ ions (FWHM 128°) comparing with that of C_3_H_5_^+^ ions (FWHM 96.75°).

### 3.4. The Angular Distribution of C_3_H_3_^+^

For quantitatively evaluating the extent of the anisotropy in the Coulomb explosion pathways and further confirm the predication mentioned above for the dehydrogenation pathway of channel (9), the expectation values of angular distribution have been defined as [23,24,25]

(Equation (4)) $$I\left(\theta \right)\propto 1+\underset{L}{\sum }a_{L}P_{L}\left(\cos\theta \right)\;(L\;=\;2,\;4,\;6)\;$$

(Equation (5)) $$\langle \cos^{2}\theta \rangle \;=\;\frac{\int I\left(\theta \right)\cos^{2}\theta \sin\theta d\theta }{\int I(\theta )\sin\theta d\theta }$$

where θ is the ejection angle of fragment ions measured from the laser polarization direction and I(θ) represents the angular distribution of the fragment ion. A_L_ is the expansion coefficients and P_L_ (cosθ) is the Legendre’s polynomials. By fitting the angular distribution of the fragment ions obtained from sliced images with the Least-square method, the expansion coefficients a_L_ and the anisotropy parameter <cos^2^θ> of channel (8) and (9) have calculated and summarized in Table 3.

For ion C_3_H_5_^+^ and C_3_H_3_^+^, the similar values of a_L_ and <cos^2^θ> indicate that the C-Br bond of the precursor molecule ions <C_3_H_3_^…^Br^…^2H>^2+^ is aligned to the laser polarization vector direction in accord with the situation in channel (8) and most of the fragment ions from channel (9) separate to each other immediately comparing with the rotation period of the precursor molecule ions <C_3_H_3_^…^Br^…^2H>^2+^, which means the dissociation lifetime of these precursor ions are much shorter than the corresponding rotation period. For a quantitative estimation of the lifetime, the Equation (4) can be written as

(Equation (6)) $$I\left(\theta ,\tau \omega \right)\;=\;1+\underset{L}{\sum }C_{L}\left(\tau \omega \right)a_{L}^{\prime }P_{L}\left(\cos\theta \right)\;(L\;=\;2,\;4,\;6)$$

(Equation (7)) $$C_{L}\left(\tau \omega \right)\;=\;\overset{L/2}{\underset{k\;=\;1}{\prod }}\frac{{\left(1/\tau \omega \right)}^{2}{+(2k-1)}^{2}}{{\left(1/\tau \omega \right)}^{2}{+(2k)}^{2}}\;(L\;=\;2,\;4,\;6)\;$$

In Equations (6) and (7), τ is the dissociation lifetime of the parent ions, ω is the rotational frequency of molecular BCP, a_L_’ is the expansion coefficients of the original angular distribution which represents the initial direction of the molecular axis just after the interaction with the intense laser field, and C_L_ is the expansion coefficients reflecting the molecular rotation prior to the dissociation as a function of τω. Assuming the C-Br bond breaking in channel (8) occurs instantaneously and the coefficients a_L_ of ion C_3_H_5_^+^ can be regarded as the original angular distribution of the molecular axis. The τω (0.216) is calculated by Equation (7).

(Equation (8)) $$\tau_{\mathrm{rot}\;}=\;\frac{2\pi }{\omega }\;=\;\frac{2\pi }{\sqrt{{\pi \mathrm{KT}(B}_{0}/\hbar^{2})}}$$

In Equation (8), τ_rot_ is the rotation period, T is the temperature and B_0_ (0.56132 cm^−1^) is the rotation constant of BCP [24]. The rotational period τ_rot_ at temperature (*T* = *100K*, the estimated temperature of super-sonic molecular beam in our experiment) is estimated to be 5.989 ps. Therefore, ω (1.049 ps^−1^) is determined. From the optimized τω values, the lifetime 206 fs of the precursor ions <C_3_H_3_^…^Br^…^2H>^2+^ can be obtained, which further confirms that direct-dehydrogenation rather than post-dehydrogenation mechanism predominates our DDI process of channel (9).

## 4. Conclusions

The Coulomb explosion and dissociative ionization of bromocyclopropane has been investigated under an intense linearly polarized femtosecond laser field by dc-slice imaging technology. The experimental results show that both DI and DDI processes are involved in the bond broken process. The ab initio calculation verifies the low KERs components of the fragments are contributed by the dissociation of singly charged parent ions. The high kinetic energy components originating from the DDI of doubly charged parent ions has been confirmed. Additionally, it is believed that the direct-hydrogenation pathway is involved in the DDI process of channel (9) and the dissociation lifetime 206 fs is much shorter than the rotation period of the related precursor molecular ions <C_3_H_3_^…^Br^…^2H>^2+^.

## Figures and Tables

**Table 1:** The kinetic energy distributions (KER), the minimum number of 800 nm photos, the transnational kinetic energy release (TER) of the corresponding photo-dissociation channel, the corresponding appearance energy and the available energy for different DI channels.

Channel	Appearance Energy (eV)	Absorbed Photon No.	KER (eV)	TER (eV)	Available Energy (eV)
(1)	10.25	7	0.173	0.26	0.60
(2)	11.46	8	0.233	0.34	0.94
(3)	12.53	9	0.107	0.48	1.42
(4)	12.66	9	0.164	0.21	1.29
(5)	14.35	10	0.239	0.27	1.15
(6)	15.28	10	0.002	0.02	0.22
(7)	16.42	11	0.146	0.43	0.63

**Table 2:** The corresponding mass ratio, the calculated KER ratio, and the experimental error Δ for different CE channels.

Channel	Ion A (mass)	Ion B (mass)	MassRatio	KERRatio	Experimental Error Factor Δ
(8)	C_3_H_5_^+^(41)	Br^+^(79)	0.519	0.540	4.0%
(9)	C_3_H_3_^+^(39)	Br+(79)	0.494	0.465	5.8%

**Table 3:** Coefficients of Legendre expansion and expectation values of the squared-cosine of the two Coulomb explosion pathways for BCP^2+.^

Channel	a_2_	a_4_	a_6_	<cos^2^θ>
(8)	1.19	−0.16	−0.03	0.49
(9)	1.05	−0.11	0.01	0.47
